# Guidewire passage through metal stent mesh using a novel sphincterotome during the stent-in-stent technique

**DOI:** 10.1055/a-2830-5068

**Published:** 2026-03-25

**Authors:** Shinsuke Akiyama, Takuya Kobayashi, Gensho Tanke, Masaya Wada, Tetsuro Inokuma

**Affiliations:** 1 26330 Department of Gastroenterology, Kobe City Medical Center General Hospital, Kobe, Japan

**Fig. 1:**
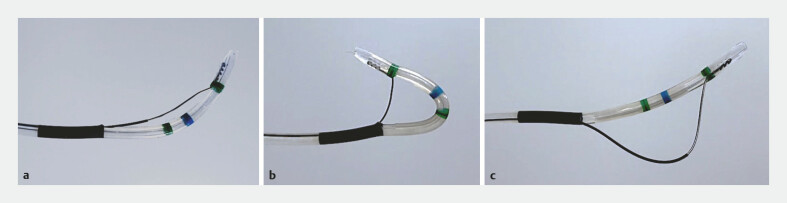
The novel sphincterotome (ENGETSU; Kaneka Corp., Osaka, Japan) has two cutting functions. **a** Normal configuration. **b** Pull configuration. **c** Push configuration.

**Fig. 2:**
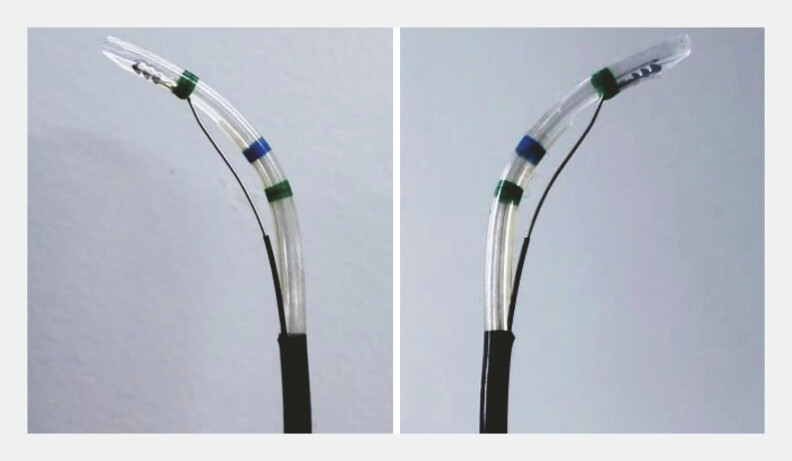
The novel sphincterotome also demonstrates superior rotational performance, enabling stepwise 180-degree rotation in both clockwise and counterclockwise directions.

The stent-in-stent technique using metal stents is widely employed for the management of perihilar biliary strictures caused by cholangiocarcinoma 1 . However, guidewire passage through the mesh of a previously placed metal stent remains technically challenging when the target bile duct axis is not aligned with the stent lumen 2 . Recently, a novel sphincterotome (ENGETSU; Kaneka Corp., Osaka, Japan) designed for endoscopic sphincterotomy has become available. Owing to its wide range of motion and rotational capability ( Fig. 1 , Fig. 2 ), this device has been reported to be effective for endoscopic sphincterotomy in patients with surgically altered anatomy 3 4 and for selective biliary cannulation, including transpapillary gallbladder drainage 5 . Herein, we report a case in which this sphincterotome facilitated guidewire passage through the stent mesh during the stent-in-stent technique ( Video 1 ).

Use of a novel sphincterotome to facilitate guidewire passage through the metal stent mesh during the stent-in-stent technique.Video 1

**Fig. 3:**
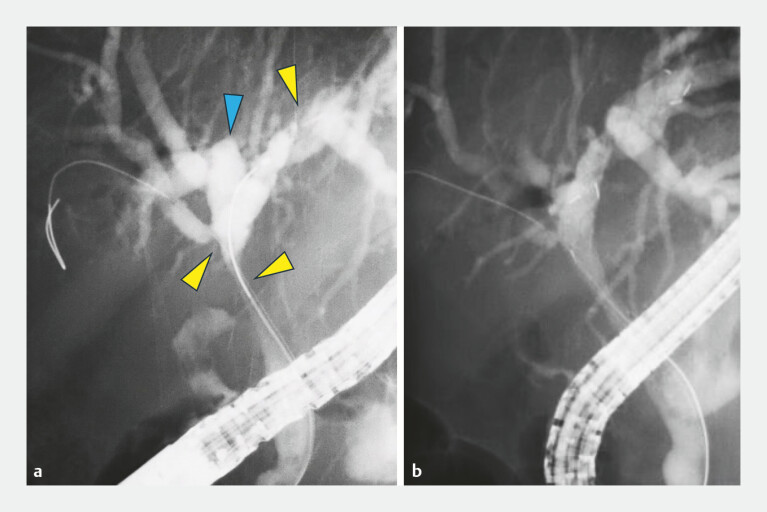
Fluoroscopic images during biliary metal stent placement using the stent-in-stent technique. **a** ERCP revealed an irregular stricture extending from the common hepatic duct to the origin of the anterior segmental branch (yellow arrowheads). An abnormality was also noted in the posterior segmental branch, originating from the left hepatic duct (blue arrowhead). **b** Initially, an uncovered self-expandable metallic stent (8 mm × 8 cm) was deployed into the B2 segment.

**Fig. 4:**
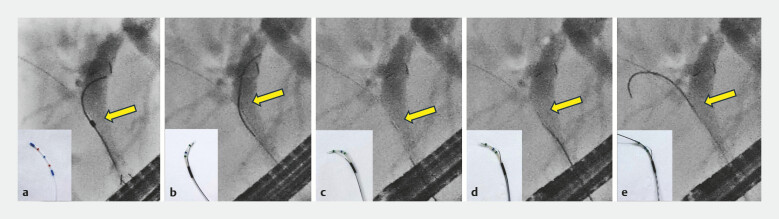
Fluoroscopic images demonstrating guidewire passage through the mesh of a previously placed metal stent during the stent-in-stent technique. Yellow arrows indicate the catheter tips of the devices used: a standard injection catheter (MTW Endoskopie Manufaktur, Wesel, Germany) and a novel sphincterotome (ENGETSU; Kaneka Corp., Osaka, Japan). **a** An initial attempt to advance a 0.025-inch guidewire through the metal stent mesh using the standard injection catheter failed because of misalignment with the axis of the anterior segmental branch. **b** The standard catheter was exchanged for the ENGETSU sphincterotome, which was initially oriented toward the left hepatic duct. **c** Under fluoroscopic guidance, counterclockwise rotation was applied to the ENGETSU. **d** A push maneuver applied to the ENGETSU blade corrected the alignment with the anterior segmental branch. **e** The guidewire was subsequently advanced successfully through the stent mesh.

**Fig. 5:**
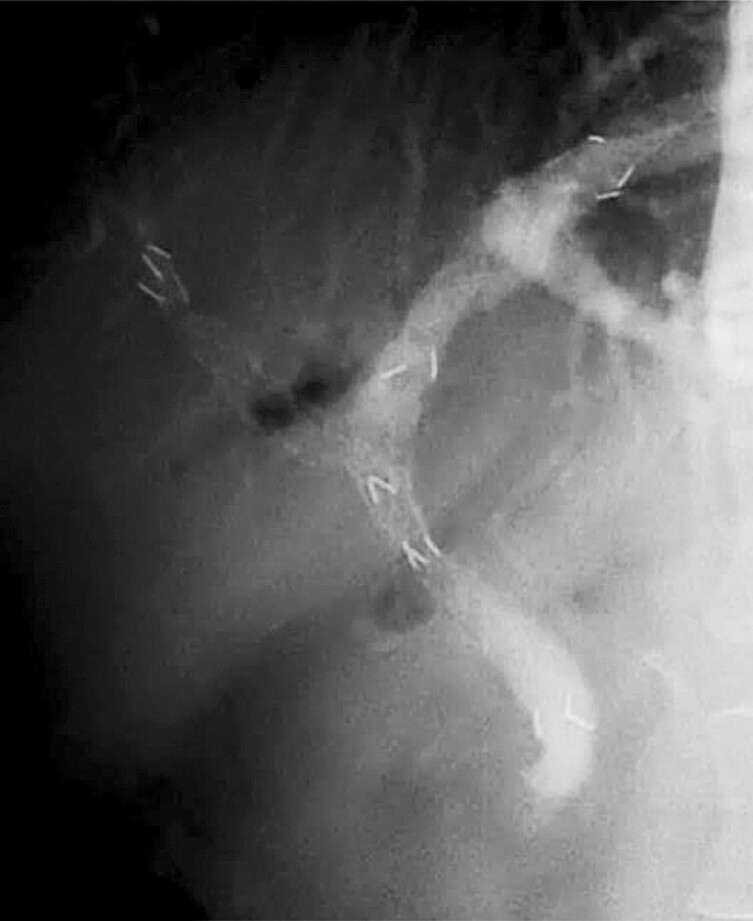
A final fluoroscopic view demonstrating successful bilateral metal stent placement using the stent-in-stent technique.

A 75-year-old man receiving chemotherapy for gallbladder cancer presented with jaundice due to occlusion of a previously placed inner stent. The stent was removed, and endoscopic retrograde cholangiopancreatography (ERCP) revealed multiple strictures involving the common hepatic duct, the origin of the anterior segmental branch, and the left hepatic duct ( Fig. 3 **a** ). Initially, an uncovered self-expandable metallic stent (8 mm × 8 cm) was deployed into the B2 segment ( Fig. 3 **b** ). Subsequent attempts to advance a 0.025-inch guidewire through the stent mesh using a standard injection catheter were unsuccessful because of misalignment with the axis of the anterior segmental branch. The catheter was therefore exchanged for the ENGETSU sphincterotome. Under fluoroscopic guidance, counterclockwise rotation combined with a push maneuver enabled successful guidewire passage through the stent mesh into the anterior segmental branch ( Fig. 4 ). Additional stent placement using the stent-in-stent technique was successfully performed, resulting in the resolution of jaundice ( Fig. 5 ).

This case highlights the usefulness of a novel sphincterotome for guidewire manipulation through the metal stent mesh in technically challenging stent-in-stent procedures.

Endoscopy_UCTN_Code_TTT_1AR_2AZ
